# Solving Classification Problems for Large Sets of Protein Sequences with the Example of Hox and ParaHox Proteins

**DOI:** 10.3390/jdb4010008

**Published:** 2016-02-04

**Authors:** Stefanie D. Hueber, Tancred Frickey

**Affiliations:** Department of Biology, University of Konstanz, Konstanz 78464, Germany; tancred.frickey@uni-konstanz.de

**Keywords:** Hox, ParaHox, Cdx, caudal, Pdx, Xlox, Gsx, Gsh, intermediate neuroblasts defective, molecular function, sequence-to-function

## Abstract

Phylogenetic methods are key to providing models for how a given protein family evolved. However, these methods run into difficulties when sequence divergence is either too low or too high. Here, we provide a case study of Hox and ParaHox proteins so that additional insights can be gained using a new computational approach to help solve old classification problems. For two (Gsx and Cdx) out of three ParaHox proteins the assignments differ between the currently most established view and four alternative scenarios. We use a non-phylogenetic, pairwise-sequence-similarity-based method to assess which of the previous predictions, if any, are best supported by the sequence-similarity relationships between Hox and ParaHox proteins. The overall sequence-similarities show Gsx to be most similar to Hox2–3, and Cdx to be most similar to Hox4–8. The results indicate that a purely pairwise-sequence-similarity-based approach can provide additional information not only when phylogenetic inference methods have insufficient information to provide reliable classifications (as was shown previously for central Hox proteins), but also when the sequence variation is so high that the resulting phylogenetic reconstructions are likely plagued by long-branch-attraction artifacts.

## 1. Introduction

One key feature of classifying protein sequences is that the classification provides the best surrogate measure for predicting likely functional properties of novel protein sequences by comparing and transferring information from better described proteins in closely related groups or clades. It is important to stress that, when focusing solely on the protein coding sequence of a gene, the resulting functional inferences for the corresponding proteins cannot take into account the expression pattern (location and concentration) and can therefore only provide information about the likely “molecular” or “biochemical” function a given protein sequence will exhibit. The underlying assumption here is that more sequence-similar proteins are also more likely to have similar biochemical properties. All functional predictions, be they based on phylogenetic or sequence-similarity analyses, only ever provide a best-guess and need to be tested experimentally.

Depending on the precise question to be resolved, different approaches to classification and prediction of protein function are used. For example, a very rudimentary prediction of protein function can be based on comparisons of domain compositions (e.g., [1]); all homeodomain-containing proteins are presumed to, at least, have the ability to bind to DNA. For those proteins with highly similar domain compositions, additional sequence features can be subsequently employed to refine the classification and prediction of protein function (e.g., see [2]). Such additional features might be the complete amino-acid sequence of a protein or, if additional structural knowledge is available, a focused analysis of the active site residues or residues known to be involved in substrate or interaction partner binding motifs.

Most protein classifications are based on phylogenetic inference methods. These are well-established standard methods aimed at answering questions by placing the data in an explicit evolutionary context (generation of an evolutionary tree of the protein family). For phylogenetic inference methods to work well, a number of conditions have to be met: The protein sequences to be classified must have evolved in accordance with the underlying model of evolution the phylogenetic inference method assumes, and the multiple sequence alignment from which the tree is inferred must be correct (or as correct as possible). Because of the latter, multiple sequence alignments are often truncated to include only unambiguously alignable regions prior to phylogenetic inference. Generating such unambiguous alignments is only possible for long stretches of highly conserved sequence regions, which can cause problems for many protein families. In the case of Hox and ParaHox proteins, the only unambiguously alignable sequence region is the homeodomain and short conserved adjacent regions. While this approach results in a robust, reproducible classification for most Hox proteins, there are limitations. The central Hox proteins (Hox4–8), for example, are so similar in their extended homeodomain that multiple sequence alignments do not provide sufficient sequence variation across these sequences for phylogenetic inference methods to resolve their relationships (reviewed in Hueber *et al*. 2010 [3]). An alternative approach was developed that is based on Profile-Hidden-Markov-Models (HMM) [4]. The advantage of HMM-based approaches is that it is possible to generate different profiles that are specific to subsets of a family. This makes it possible to include some regions that are conserved in a specific sub-group, but that do not need to be present or conserved across all groups. The HMM profile approach, while very fast and useful for verifying to which Hox protein group a specific sequence may belong in general (checking for mis-annotations), did not help resolve the relationships between vertebrate and arthropod central Hox proteins. This is not surprising as, even with this approach, not all sequence regions can be included. A multiple sequence alignment provides the basis for the derived HMM's and, ideally, only unambiguously alignable regions should be employed. The quality of any multiple alignment-based classification will also greatly depend on the skill of the researcher generating the multiple alignment, as human perception and experience can increase alignment quality and human, computational or sequence biases can decrease it.

Advances in experimental and computational resources provide us with access to a multitude of tools and data, as well as the computational power to make better sense of the biological data we generate. So far, the only method that successfully classified the central Hox proteins based on sequence information employed a fairly recently developed method called CLANS [5]. CLANS is based on all-against-all pairwise sequence similarities and thus circumvents the need for an multiple alignment entirely. The CLANS-based approach uncovered that Hox7 and Antp protein sequence-similarity groups are conserved throughout all bilaterian clades, and that the Hox6 and Hox8 protein groups are specific to the vertebrate lineage and are markedly different from the most similar relatives present in the chordate amphioxus. It also detected two new groups of central Hox proteins, termed Echi/Hemi 7 and Echi/Hemi 8 [6].

**Figure 1:**
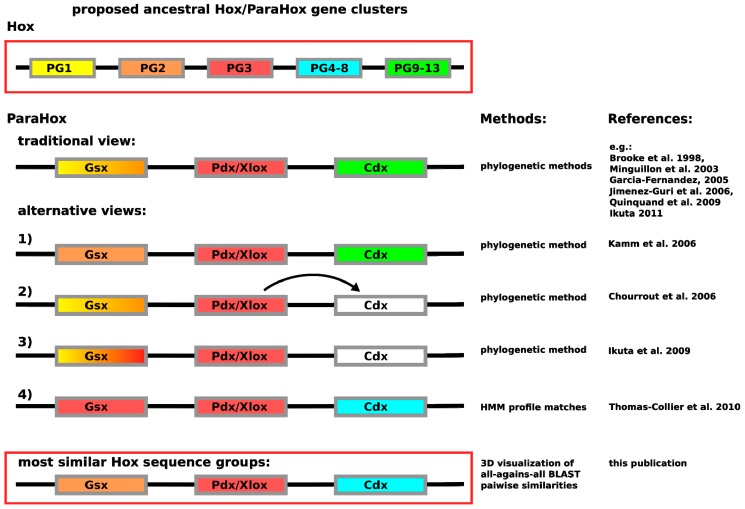
Depiction of the currently proposed relationships between *ParaHox* and *Hox* genes. Similar colors between *Hox* and *ParaHox* reflect the different assignments. The traditional view proposes that the ancestral *ParaHox* cluster contained genes whose proteins corresponded to at least one anterior (Hox1/2), one Hox3 and one posterior (Hox9+/Abd-B) type Hox protein [7,8,9,10,11,12]. Alternative views propose slightly different scenarios, except for the classification based on hidden Markov models in which Gsx is assigned to the Hox3 group and Cdx to the central Hox protein group (Hox4–8) [4,13,14,15].

In contrast to having the problem of insufficient sequence variation across the homeodomain, the Hox/ParaHox field has likely encountered the opposite problem. So far, a number of different predictions are available for how the respective Hox and ParaHox genes are thought to have evolved from an ancestral Ur-*Hox*/*ParaHox* gene or gene-cluster. The most commonly depicted view is that Gsx and Hox1/2, Pdx/Xlox and Hox3 as well as Cdx and the abdominal Hox proteins (Hox9–13) are likely to have been derived from the same genes in the ancestral *Hox*/*ParaHox* gene-cluster (see Figure 1). While the assignment of Pdx/Xlox sequences as sequences most closely related to Hox3 seem to be consistent, no matter which method is used, the assignment of other ParaHox proteins to prospective Hox counterparts varies. For Gsx the assignments vary: the traditional view of Hox1–2 [7,8,9,10,11,12], a further undefined Hox1–3 group [13], a clear assignment of Hox2 based on the depicted tree [14] or a specific assignment of Hox3-like sequences as close relatives [4]. The assignments of Cdx in the literature, in contrast, differs from the commonly depicted view (see Figure 1) only in such a way that Cdx either cannot be assigned as closely related to any specific group, or that authors with similar data interpret Cdx as derived from Pdx/Xlox [13,15]. The only analysis providing a noticeably different result is the HMM-based analysis carried out by Thomas-Chollier *et al.* [4]. In this case, Cdx was assigned as most likely to be a Hox4–8 derivative. What can cause such a drastic difference in assignment between HMM-based and phyogenetic-based methods? Considering that the posterior Hox proteins are known to be highly divergent in their sequence composition [3,16], it is possible that the ParaHox *vs.* Hox classification field is suffering from a phenomenon called long-branch attraction (LBA). In LBA, systematic classification artifacts are generated due to too much variation in a subset of the aligned sequences. To make the scenario for LBA likely would, however, require Cdx as well as abdominal Hox proteins to be highly divergent in their sequences when compared to other Hox and ParaHox proteins.

To gain a different, non-multiple-alignment-based view of the relationships between these sequences, we used the same approach that resolved the relationships of central Hox proteins across bilaterians. Here, we show that this approach can improve classifications not only when multiple sequence alignment-based methods have insufficient information to provide reliable classifications, but also when the sequence variation is so high that it induces systematic artifacts in the resulting phylogentic reconstructions (see variations in assignment of Cdx in Figure 1), in this example, there is a putative long branch attraction (LBA).

## 2. Experimental Section

**Figure 2:**
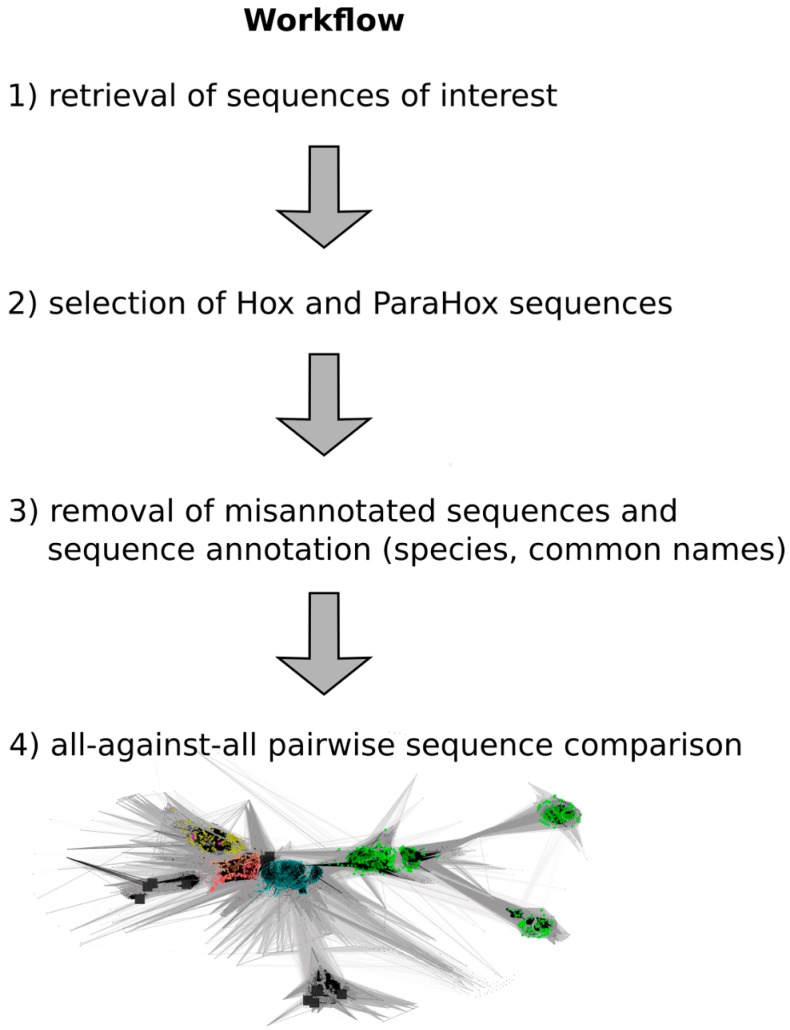
Work flow depicting the major steps required to generate a full-length Hox/ParaHox all-against-all sequence comparison CLANS file. (**1**) To retrieve the sequences of interest, a homeodomain search against the NCBI non redundant protein database was carried out; (**2**) Sequences belonging to the groups of Hox and ParaHox proteins were identified and extracted based on a first sequence-similarity map; (**3**) As some "Hox" sequences were apparent mis-annotations, we removed those sequences from the dataset (e.g., sequences that consisted of a concatenation of multiple different Hox proteins), and the remaining sequences were further annotated by appending common names and the species the sequences originated from; (**4**) The final step was the visualization and analysis of the all-against-all pairwise sequence similarities for the group of Hox and ParaHox sequences in 2D and 3D space.

A flow chart of the approach is depicted in Figure 2, and a detailed step-by-step guide of the approach is available in the Supplementary Materials as “Approach.pdf”.

Similar to the approach described in Hueber *et al.* 2010 and Hueber *et al.* 2013 [3,6], we searched the NCBI non-redundant protein database (non redundant = “nr” downloaded 16 September 2015) for all sequences containing a Hox/ParaHox-like homeodomain. The alignment we based our search strategy on is available in the Supplementary Materials as “HD62.aln,” the original unaligned sequences as “seedsequences.txt.” The alignment was generated using MUSCLE (version 3.7-r1) and manually curated in AlnEdit. A Profile-Hidden-Markov-Model was derived from this alignment using HMMER version 3.0 [17]. This HMM was used to search the non-redundant protein database for sequences resembling our set of Hox/ParaHox proteins. For this set of sequences, we then generated a pairwise-sequence-similarity map using CLANS, a pairwise-sequence-similarity visualization tool (which allows visualization in both a 2D and 3D space—the 3D graphs provide more visual information to the viewer, but are harder to incorporate into a printed manuscript).

To generate the all-against-all pairwise sequence-similarities, Blast version 2.2.30 was used (downloaded on 8 October 2014) [18] as part of the CLANS program [5]. The precise version used to perform the all-against-all pairwise sequence comparison is provided in the Supplementary Materials (clans.core.jar). Based on the resulting sequence-similarity-maps generated by CLANS, sequence clusters containing Hox and ParaHox sequences were selected. As mis-annotated Hox/ParaHox sequences containing multiple homeodomains were detected, we identified those sequences containing more than a single 60 amino acid homeodomain using HMMER version 2.3.2 and excluded them from the analysis. In addition to the already present unique sequence identifier (gi number) and the sequence itself, the remaining sequences were further annotated with common names, e.g., Hox1–13 or Lab-AbdB, as well as the species the sequences originated from.

The Supplementary Materials as “Approach.pdf” provides specific details and step-by-step instructions to reproduce the above analysis.

As the nomenclature for the proteins we are working with can vary between organisms (due to tradition or due to mis-annotations), we would like to explicitly state that for deuterostomes we employ the vertebrate nomenclature (using *Mus musculus* as a standard), and for protostomes we employ the nomenclature based on *Drosophila melanogaster* (which only has two ParaHox protein types) and *Capitella teleta* (which has all three ParaHox protein types) as a standard for annotating the resulting cluster groups.

## 3. Results and Discussion

In situations where different variants of an approach yield varying results, the use of an independent method may provide additional insights. In this case we opted for a comparison of all-against-all pairwise sequence similarities of full-length Hox/ParaHox sequences to shed light on the classification of ParaHox proteins in relation to Hox-protein groups. The aim of this method is to avoid the introduction of user-based biases induced by generating and analyzing user-defined multiple sequence alignments (we use the alignment and HMM-searches only to retrieve all sequences of interest and then carry out the analysis with unaligned sequences). In addition, as there is not much information available on how precisely ParaHox proteins carry out their molecular functions and what sequence elements precisely are relevant to their function, we decided to exclude potential user-induced (Hox-research focused scientists) biases and analyze the full-length protein sequences rather than focusing on specific domains or subsections of these proteins.

**Figure 3:**
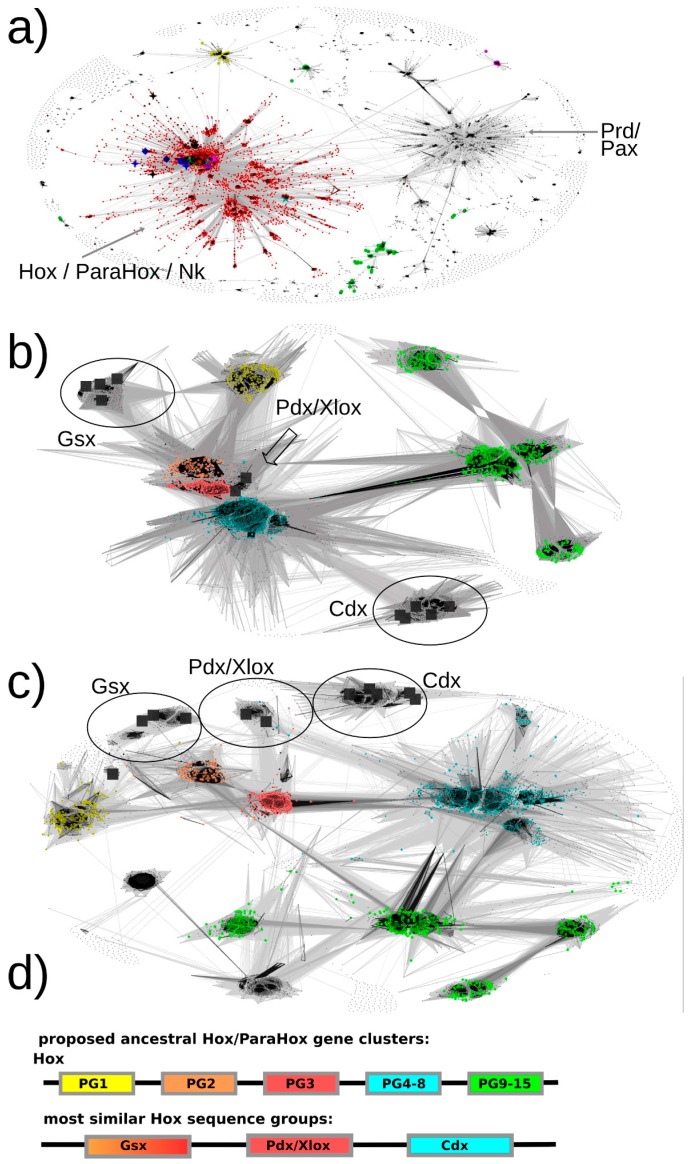
All-against-all pairwise-sequence-similarity maps of full-length protein sequences mapped in 2D. Each sequence is represented by a dot in the 2D space. The lines connecting the dots represent pairwise sequence similarities. The darker the lines, the more significant (lower) the E-value of the reciprocal blast hits. (**a**) The sequence-similarity map of all sequences of potential interest to us (see methods, step 7) with the red dots representing the selected Hox/ParaHox/Nk cluster. (**b** + **c**) All sequences of the Hox/ParaHox cluster, with (**b**) depicting the map at an E-value cutoff of 1e-33 and (**c**) depicting the map at an E-value cutoff of 1e-40. Yellow dots represent Hox1/Lab sequences, orange dots Hox2/Pb, red dots Hox3, blue dots central Hox proteins (Hox4–8/Antp/Abd-A/Ubx) and green dots represent posterior Hox proteins (Hox9+/Abd-B). The grey squares represent the ParaHox proteins from *Mus musculus*, *Drosophila melanogaster* and *Capitella teleta*. (**d**) Summary of which ParaHox proteins are most similar to which Hox groups. Please note that the coloring only represent the observed highest similarity in sequence across the respective groups and is not meant to provide a prediction of functional similarity (see text for more details).

Our basic approach to identifying Hox/ParaHox proteins is likely to have ensured a full inclusion of all Hox and ParaHox protein sequences as we retrieved plant TALE homeodomain sequences as well as Pax sequences, which are well beyond the stated scope of this study. As a first step, we generously selected all sequence-similarity clusters containing one or more of our seed sequences (Figure 3a, red dots). For the second step, we extracted these sequences to a separate file, re-inflated the dataset by reversing the CD-hit filtering (as described in the Supplementary Materials methods section) and removed known non-Hox/ParaHox sequences (e.g., NK) in order to provide us with all sequences available for our groups of interest. For the resulting set of sequences, the similarities of each ParaHox group to the most similar Hox protein group were analyzed both in 2D and 3D space (the figures here can only depict 2D).

The default map, *i.e.* the map that provided us with the best resolution for the ParaHox/Hox analysis, was calculated at an E-value cutoff of 1e-33 using sequence similarities calculated over full-length sequences.

The results are as follows (see Figure 3b,c).

### 3.1. Pdx Is the ParaHox Protein Most Similar to Any of the Hox Protein Groups and, Across Hox Proteins, Most Similar to Hox3

Based on the sequence-similarity map in Figure 3b, Pdx sequences appear very similar to Hox2/3 sequences and cluster in close proximity. To assess whether there is a sequence-similarity signal present that might allow us to further resolve the placement of Pdx, a more stringent E-value cutoff was selected and the dataset re-clustered. At an E-value cutoff of 1e-40, a greater number of high-significance sequence similarities could be observed between Pdx and the Hox3 paralog group than to any other group (see Figure 3c). These results are consistent with all previous analyses we are aware of (reviewed in [12,19]).

### 3.2. Gsx Is Similar to Hox2 and 3

As visible in Figure 3b, the sequence-similarity data indicates a clustering of Gsx group proteins with proteins from the Hox paralogy groups 2 and 3. There are also some individual proteins showing noticeable similarity to the central group Hox proteins (defined as PG4–8) (mainly to the Dfd/Hox4 group) as well as to the Hox1 group. However, the most consistent signal (number of connections and darkest lines) indicates Gsx proteins, as a group, being most similar to Hox2 and Hox3 proteins.

Gsx has previously either been assigned as similar to Hox1 or Hox2 (with some exceptions assigning it to Hox2 alone, Hox3 alone or Hox1-3-like) (Figure 1). Our data does not support the notion of a higher similarity being present between Gsx and Hox1 proteins, but rather support an assignment to Hox2 and Hox3 (both Hox2 and Hox3 are also more similar to each other than to Hox1). Using more stringent E-value cutoffs up to 1e-40 (at which point most connections are lost), we also examined whether there is a signal in the sequence-similarity data preferentially assigning Gsx to either Hox2 or Hox3. However, the connections seem roughly equal in number and quality, which indicates that, at least for full-length sequences, the sequence assignment of Gsx to being either more Hox2 or more Hox3-like cannot be resolved using this approach.

### 3.3. Cdx Is Most Similar to the “Central-Group” Hox Proteins

Previous methods either assigned Cdx to the posterior Hox protein group (Hox9–13/Abd-B) or did not provide a clear assignment due to insufficient resolution or conflicting data. In contrast, our sequence-similarity map clearly indicates a much higher similarity of Cdx group proteins to the central-group Hox proteins than to any other group of Hox proteins. In addition, it should be noted that the Cdx- and Hox-posterior-group proteins are located the most distant from the center of the map, indicating a greater than average dissimilarity in sequence than the rest of the Hox/ParaHox sequences. The distance from the center and absence of connections between the Cdx-group and any of the Hox-posterior-group sequences further supports the idea that these two groups of proteins have accumulated more changes in sequence than the other Hox/ParaHox proteins and indicates that they have done so independently of each other.

Assuming a higher than average rate and independence of accumulated mutations for these groups, helps explain the discrepancy between our results and the claim that Cdx proteins are similar to the posterior-type Hox proteins. Fortunately, the literature also indicates that this claim is not universally accepted. One publication depicts the sequence deviation of Cdx from other Hox and ParaHox proteins in an unrooted tree (and, perhaps because of this, even suggested that the *Cdx* gene might have been independently duplicated in the *ParaHox* gene-cluster), another publication used a different method and actually stated that there is a similarity of Cdx to central Hox proteins [4,15]. The latter result, however, seems to have gone unnoticed by recent reviews [12,19]. One explanation for the difficulty in achieving a consistent classification of Cdx and posterior-group Hox proteins in relation to the other Hox/ParaHox proteins using phylogenetic approaches lies in the notorious long-branch-attraction (LBA) artifact. The LBA-problem is especially prevalent when groups of highly similar proteins are being compared to much more dissimilar proteins, as classification methods have a tendency to group the similar sequences together, thereby leading to an artificial, exclusion-based, grouping of the more dissimilar sequences. If this effect is not considered and corrected for, it can lead to serious artifacts in phylogenetic reconstructions. Based on the sequence-similarity data available to us, we would postulate that LBA-effects are likely to have contributed to the differences observed between previous attempts at classifying these protein families

## 4. Conclusions

A number of hypotheses, with varying degrees of support, are present in the current literature to explain how the *Hox* and *ParaHox* genes arose from an ancestral UR-*Hox*/*ParaHox* gene or gene-cluster (Figure 1). An UR-*Hox*/*ParaHox* gene must have duplicated or triplicated to give rise to the proto-*Hox*/*ParaHox* gene-cluster. The number of suggested proto *Hox*/*ParaHox* genes, however, varies between 2 and 4 [19]. The most commonly depicted view is that there must have been at least three genes, one anterior *Hox*1/2/*Gsx* like, one *Hox*3/*Xlox*/*Pdx* like and one *Cdx*/*Hox*9+ like (see Figure 1) [12]. This gene-cluster then duplicated giving rise to the *ParaHox* and *Hox* clusters, the latter of which further duplicated some of its constituent genes.

Using a method analyzing pairwise sequence similarities across all available Hox/ParaHox full-length protein sequences provides us with more information.

While our results support the hypothesis that Pdx/Xlox and Hox PG3 proteins are encoded by genes that arose from the same ancestral gene in the proto *Hox*/*ParaHox* cluster, the assignments of Gsx to Hox1–2 and Cdx to abdominal class Hox proteins (PG9+/Abd-B), however, cannot be supported and may have to be reevaluated. Previous analyses suggested Gsx being similar to Hox1–3, in varying combinations (see Figure 1). While Gsx does share similarity with Hox1, it does so to the same degree as to central Hox proteins. By far the strongest sequence-similarity signal is to the group of Hox2/3 proteins. This result allows us to postulate that the corresponding *Hox*2/3 and *Gsx* genes are most likely to have diverged from a common gene in the ancestral *Hox*/*ParaHox* gene-cluster (and that other pairing are less likely), a result similar to, yet slightly different from, previous analyses. As for Cdx, a consistent higher sequence similarity to central group Hox proteins (here defined as PG4–8) than to any other group of Hox proteins (including PG9+/Abd-B) indicates that the central Hox and Cdx protein coding genes arose from the same ancestral gene in the proto *Hox*/*ParaHox* cluster. A simple explanation as to why some previous analyses assigned Cdx to posterior Hox lies in their increased difference in sequence when compared to all other Hox/ParaHox sequences. This increased sequence dissimilarity can cause some methods to suffer from the long-branch-attraction artifact, causing the least similar sequences to artificially group together in a tree.

In terms of the number of proto ParaHox/Hox proteins that must have been encoded in the gene-cluster before the spit into *Hox* and *ParaHox* gene-clusters, we can see evidence of at least two different protein types: Hox2/3/Gsx/Xlox/Pdx and central Hox/Cdx. Whether Gsx/Hox2–3 and Xlox/Pdx/Hox3 may have been originally encoded by two different genes (meaning the proto *ParaHox*/*Hox* cluster contained three genes in total) or by a single gene with *Xlox*/*Pdx* representing a gene duplication from *Gsx* (or vice versa), we cannot say given the current data.

In terms of how far we can expect Hox and ParaHox proteins to show a similarity in their molecular function (e.g., DNA and protein binding abilities and preferences), it seems plausible that Pdx/Xlox will exhibit similar molecular functions to Hox3 proteins in roughly the same way that Hox2 proteins behave similarly to Hox3 proteins (we cannot yet quantify how similar that is, as we do not have any quantitative comparative experimental data available). Considering the high sequence divergence of Cdx, we would presume Cdx to behave significantly differently from all other Hox and ParaHox proteins. Somewhere in between these two extremes is Gsx. Since Hox2 and 3 are more similar to each other than they are to Gsx, it is possible that Gsx differs significantly in a number of molecular functions. We suggest that, in cases where Gsx does act similarly to Hox2/3, whether Gsx will act more like a Hox2 or more like a Hox3 protein will depend on the precise sequence region involved in a given molecular function.

No other set of transcription factors provides us with a set of sequences that are as similar across regions known to be relevant to protein function (e.g., the DNA binding homeodomain), and yet whose members induce such wildly different effects, even when expressed in the same concentration, location and space. If we ever want to fully understand precisely how homeodomain transcription factors (and perhaps other transcription factors) achieve their specific function, the set of Hox and ParaHox proteins is probably the best set of proteins to study. One first step is to identify linear motifs [20] and figure out what each putative motif contributes to the protein function. Another way in which the Hox/ParaHox field should excel is an expansion of the existing “top-down” approaches of generating rescue experiments, as well as comparative and quantitative data of phenotypes or expression data for Hox and ParaHox proteins (e.g., [21,22,23,24,25,26]). This expansion of our traditional “top down” approaches could be done by including comparable analyses of the molecular functions of the proteins, e.g., qualitative and quantitative protein-protein and protein-DNA interaction studies, in order to establish a thorough overview of which sequence regions of the proteins are involved in achieving which molecular functions.

## Supplementary Materials

The following are available online at www.mdpi.com/2221-3759/4/1/8/s1, Files: Approach.pdf, seedsequence.txt, HD62.aln, clans.core.jar, ParaHox_CLANS_sequences.txt, perl (folder), reference.txt. The Supplementary Materials above as well as the Clans file used for the analysis are also available for download: http://bioinformatics.uni-konstanz.de/HueberHox/ParaHox/.
