# Supplementary material for: Solving Classification Problems for Large Sets of Protein Sequences with the Example of Hox and ParaHox Proteins
Source: J Dev Biol. 2016 Feb 4;4(1):8. doi: 10.3390/jdb4010008 (PMC5831817; doi:10.3390/jdb4010008)
Supplement: Supplementary file 1 [file jdb-04-00008-s001.zip › jdb-109482-supplementary - for windows.zip!/Hueber_2016_ParaHox_supplementary_files_JDB/Approach.pdf]

Detailed Approach of Large protein families and what we can learn from Hox and ParaHox

(Stefanie D. Hueber and Tancred Frickey, JDB, 2016)

We used full-length sequences of all Hox and ParaHox proteins available in the Flybase database [9], downloaded on 10th Sept. 2015. For each protein, the isoform with the longest amino-acid sequence was chosen as the representative sequence. The corresponding set of sequences is available in the supplementary materials section (seedsequences.txt). As the nomenclature for the proteins we are working with can vary between organisms (traditionally or due to mis-annotations), we would like to explicitly state that, for deuterostomes, we employ the vertebrate nomenclature (using Mus musculus as a standard) and, for protostomes we employ the nomenclature based on Drosophila melanogaster (which only has two ParaHox protein types) and Capitella teleta (which has all three ParaHox protein types) as a standard for annotating the resulting cluster groups. A flow-chart of the approach is depicted in Figure 2.

step 1: retrieving sequences of interest

The NCBI non-redundant protein database ('nr' downloaded 16.09.2015) [10] and associated files provide our starting point to identify all sequences we might be interested in for this analysis. The reference sequences extracted from Flybase, see above, were first multiply aligned using MUSCLE version 3.7-r1 [11] and the resulting automated alignment was manually curated using the alignment editor AlnEdit [12], placing a focus on high-quality alignment of the homeodomains of these protein sequences. This set of aligned reference sequences is available in the appendix as file HD62.aln. Next, a Profile Hidden Markov Model (HMM) was derived from this multiple alignment, using HMMER version 3.0 [13], and used to search the NCBI 'nr' database for sequences resembling our set of Hox/ParaHox proteins. As the aim of this step was to retrieve a most encompassing set of sequences, HMMER version 3.0 was used.

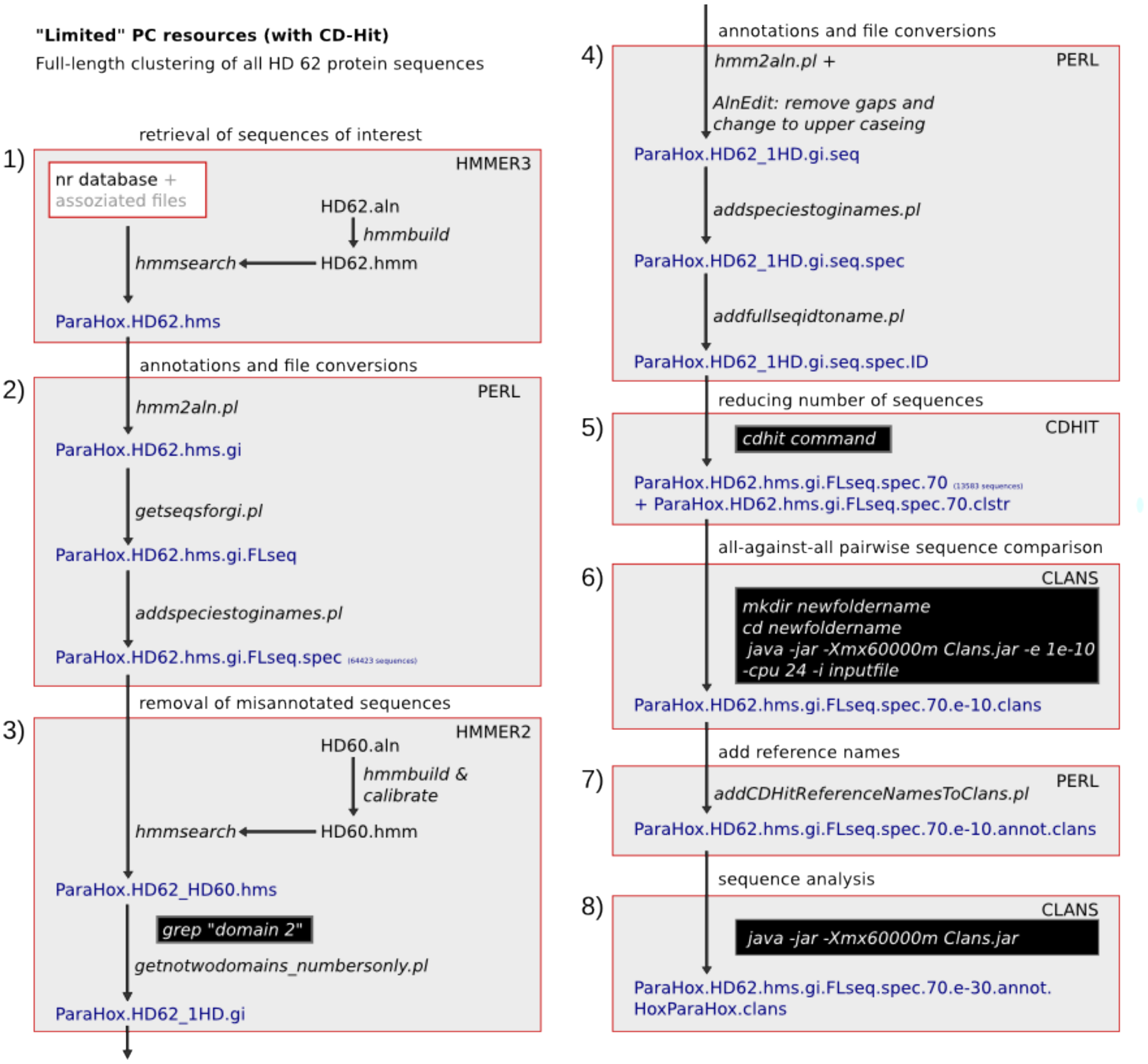

Figure 2. Work-flow for generating the full-length Hox/ParaHox all-against-all sequence comparison CLANS file. “Limited” computational resources required us to use CD-Hit at early stages of the work flow to reduce the amount of data to be analyzed to a manageable size. Once large amount of data could safely be excluded from the analysis, the CD-Hit step was reversed to include and visualize all Hox and Hox/ParaHox like sequences available in the NCBI non-redundant protein database.

step 2: annotation and file conversions

All perl scripts used as part of this analysis are made available in the supplementary materials. The first script, hmms2aln.pl, takes the HMM search output and converts it into a multiple alignment format. The following scripts, getseqsforgi.pl and addspeciestoginames.pl, are used to retrieve the full-length sequences of all hits in the HMM file from the NCBI 'nr' database and add a consistent species identifier to the sequence name (based on the files 'names.dmp' and 'gi_taxid_prot.dmp', both provided by NCBI). At this stage our dataset of interest encompassed 64423 potential Hox/ParaHox sequences.

step 3: removal of misannotated sequences

In the current NCBI database, some entries can be found that contained multiple homeodomain proteins concatenated into a single sequence (e.g. Hox2 fused to Hox3 and Hox4). Since all Hox and ParaHox protein sequences that were studied to date always only contained a single homeodomain, these sequences likely represent mis-annotations or artificial constructs. To remove such sequences from our dataset we generated an alignment of the 60 amino acid homeodomain (as defined by Gehring [14]) in the same manner as described in step 1, with one key difference: for building, calibrating and searching, HMMER version 2.3.2 was used (HMMER 2 proved better at detecting the entire sequences on which the HMM was built)[15]. The results of the HMM-search against our dataset of interest (step 2) provided us with the information which of the sequences contained two or more homeodomains. Using the grep command (#more hmsfilename | grep “domain 2” >outputfilename) we identified which of the sequences had more than one homeodomain and these sequences were then removed from our set of interest using the script getnotwodomains_nubersonly.pl. The remaining dataset contained 63142 sequences/gi-numbers.

step 4: annotation and file conversions:

Similar to step 2, the full-length sequences and species names for the remaining 63142 sequences had to be retrieved from the NCBI nr database and associated files. To ensure all information remained available, the script addfullseqidtoname.pl was used to append the sequence descriptions present in the NCBI nr database to the corresponding entries.

Step 5: reducing the number of sequences (CD-Hit)

To reduce the set of 63142 sequences to a more manageable size, the program CD-hit version 4.6 was used. The dataset was filtered at a 70% identity cutoff (sequences with >=70% identity are identified as a similarity cluster and only one representative sequence for the cluster is returned), thereby reducing redundancy and the dataset size to 13538 sequences.

Step 6: all-against-all pairwise sequence comparison

To generate the all-against-all pairwise sequence-similarities, Blast version 2.2.30 was used (downloaded on 08.10.2014) [16] as part of the CLANS program [17], a pairwise-sequence-similarity visualization tool (which allows visualization in both a 2D and 3D space - the 3D graphs provide more visual information to the viewer, but are harder to incorporate into a printed manuscript). The precise version used to perform the all-against-all pairwise sequence comparison is provided in the supplementary materials (clans.core.jar) (an updated version from April 2013 also allows visualization/highlighting of all sequences from NCBI taxonomic groups (Hueber et. al 2013)). Instructions on how to use Clans are available in the corresponding manuscripts.

Step 7: add reference names

Filtering the dataset using CD_hit at a 70% identity cutoff removed some of our Hox/ParaHox reference sequences from the dataset. The script addCDHitReferenceNamesToClans.pl was used to append the name of our original reference sequences (reference.txt) to the corresponding cluster representative sequence selected by CD-hit.

Step 8: All-against-all pairwise sequence comparison and selection of the clusters of interest

As described in step 6, all-against-all sequence comparisons were performed and the resulting cluster map was used to identify sequences belonging to the Hox/ParaHox protein families. After the Hox and ParaHox-like sequence groups were identified (e-value cutoff of e-30), the >=70% identity CD-hit filtering step was reversed.

Step 9: reverse CD-Hit

CD_Hit generates two output files: an output file with sequences in fasta format and a *.clstr file, providing information about what sequences in the output file represent what groups of highly similar sequences from the input dataset. After identifying the sequence groups of interest (step8) and excluding all other sequences, the script reverse_cdhit.pl allowed us to add back to our datasets, for the remaining sequences, the individual sequences that had been removed by the CD-Hit filtering step. The output is a list of gi numbers, which means steps 4 (annotation and file conversion) and 6 (all-against-all pairwise sequence comparison) had to be repeated. After these steps, the resulting dataset is assumed to provide a thorough and high-quality sampling of the NCBI 'nr' database for sequences of the Hox/ParaHox type (11804 sequences).

The Clans file used for the analysis and the supplementary materials are available at:

http://bioinformatics.uni-konstanz.de/HueberHox/ParaHox/

(login: jdb password: parahox – all in small letters. The login and password requirements will be removed upon publication in JDB).
